# Solid/trabecular subtype of papillary thyroid carcinoma on cytology with focal differentiated high-grade thyroid carcinoma on histology: a cyto-histologic correlation

**DOI:** 10.3332/ecancer.2023.1587

**Published:** 2023-08-10

**Authors:** Ashutosh Rath, Shailaja Prabhala, Shrinivas Bheemrao Somalwar, Immanuel Pradeep, Namit Kant Singh

**Affiliations:** 1 Department of Pathology and Laboratory Medicine, All India Institute of Medical Sciences Bibinagar, Hyderabad 508126, Telangana, India; 2 Department of Otorhinolaryngology, All India Institute of Medical Sciences Bibinagar, Hyderabad 508126, Telangana, India; a https://orcid.org/0000-0001-6255-8286

**Keywords:** solid/trabecular subtype, papillary thyroid carcinoma, differentiated high-grade thyroid carcinoma

## Abstract

Solid/trabecular subtype of papillary thyroid carcinoma (S/T PTC) is a rare entity that has been shown to have higher tumour recurrence and mortality rates. A definite diagnosis on fine needle aspiration cytology is often not easy. Rather, this entity may be misdiagnosed in cytology due to a lack of widespread features of classic PTC. We present a case of S/T PTC in a 61-year-old female, showing a focus on differentiated high-grade thyroid carcinoma (DHGTC) on histology. We discuss cytological features with the histologic correlation of S/T PTC and briefly discuss the newly introduced entity, DHGTC.

## Introduction

Papillary thyroid carcinoma (PTC) is the most common thyroid malignancy. Of all the histologic subtypes, tall cell, columnar cell, and hobnail subtypes of PTC have been considered aggressive by American Thyroid Association [1]. The literature on the outcomes of solid/trabecular subtype of PTC (S/T PTC) is conflicting, with some studies reporting it as a clinically aggressive subtype [2–4]. A recent systematic review with meta-analysis of this entity has concluded that S/T PTC is an aggressive subtype with higher tumour recurrence and mortality rates [5]. Being a rare thyroid tumour, recognising S/T PTC on cytology may help clinicians understand the probable prognosis and better patient management. The fifth edition of the World Health Organisation Classification of endocrine tumours (WHO) has introduced a category of thyroid tumours called differentiated high-grade thyroid carcinoma (DHGTC), which arises from papillary or follicular patterned thyroid carcinoma with mitotic count ≥5/2 mm2 and/or necrosis [6]. We describe cytological features of a case of S/T PTC in a 61-year-old female with a discussion of histologic correlation. We also bring out the high-grade features of this subtype seen in histology with a brief literature review on this aspect of the tumour.

## Case report

**Figure 1.**
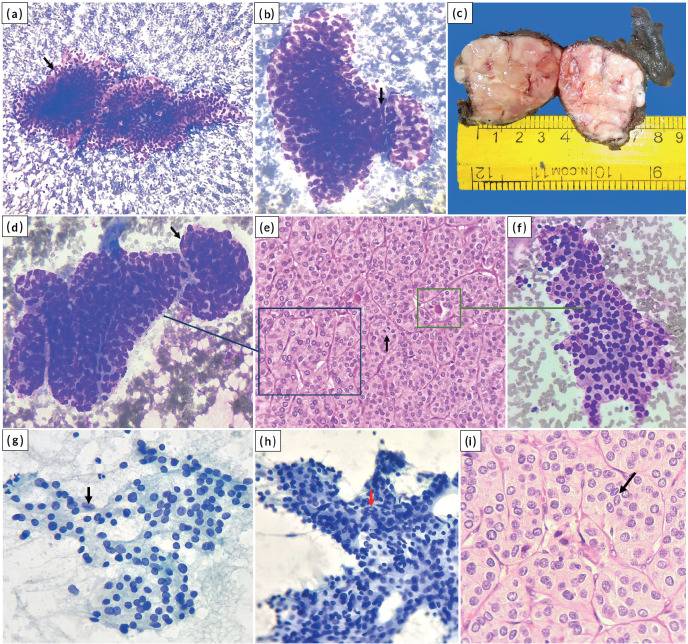
(a and b): Trabeculae and solid nests of tumour cells with focal endothelial lining (arrow) (MGG stain, (a) ×100; (b) ×400); (c): Gross image of total thyroidectomy specimen showing the solid tumour in right lobe of thyroid; (d to f): Cyto-histologic correlate; the trabecular pattern in cytology (d) resembles a similar pattern on histology (e) and focal microfollicular pattern in cytology (f) corresponds to abortive follicles on histology. An endothelial cell lining the trabecula is shown (arrow) in (d). Mitosis is shown (arrow) in (e). ((d), (f) MGG stain, ×400; (e) HE stain, ×400); (g to i): Nuclear grooving (black arrows) and INCI (red arrow) ((g), (h) Pap stain, ×400; (i) HE stain, ×400).

**Figure 2.**
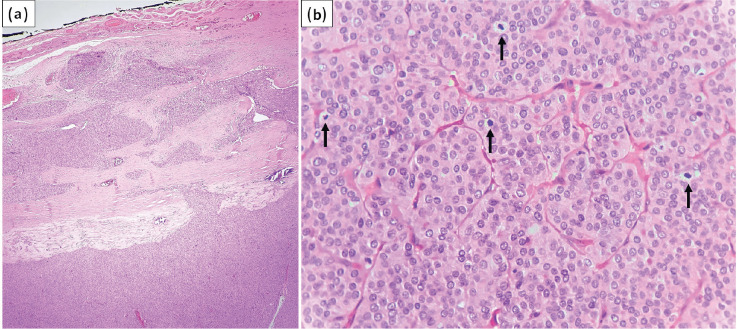
(a): Tumour exhibiting capsular invasion (HE stain, ×40); (b): Focus of DHGTC with four mitotic figures in single HPF (arrows) (HE stain, ×400).

A 61-year-old female presented with a gradually progressive, firm, 4 × 3 cm swelling in the thyroid region for 3 years. There was no history of childhood radiation or family history of thyroid carcinoma. The patient was clinicobiochemically euthyroid. Ultrasonography of the neck revealed a well-defined, heterogeneous, solid lesion in the right lobe of the thyroid measuring 4 × 3.5 × 2.5 cm. The lesion showed multiple foci of peripheral calcification and was categorised TI-RADS 4 as per thyroid imaging reporting and data system. Fine needle aspiration of the swelling was performed with two passes. The May–Grunwald–Giemsa-stained smears were moderately cellular and showed atypical follicular cells arranged in cohesive trabecular clusters, solid nests and occasional microfollicles (Figure 1a–f). The follicular cells showed nuclear crowding and overlapping with oval nuclei. Focal nuclear grooving and an occasional intranuclear cytoplasmic pseudoinclusion (INCI) were noted in Papanicolaou-stained smears (Figure 1g and h). An impression of ‘suspicious for PTC’ was rendered as per the Bethesda system for reporting thyroid cytopathology due to the lack of widespread features of conventional PTC. Consequently, a total thyroidectomy was performed. Lymph node exploration did not reveal any significant lymphadenopathy. Grossly, the cut surface of the right lobe showed a capsulated, beefy pink, fleshy, completely solid nodular growth occupying almost the entire lobe and measured 4 × 3.8 × 3 cm (Figure 1c). The sections revealed a diagnosis of S/T PTC with nearly the whole tumour in a trabecular/nested pattern (Figure 1e and i). Papillae and tumour necrosis were not seen. Extensive capsular invasion and focal vascular invasion (<4 vessels) were seen (Figure 2a). Extrathyroidal extension was not noted. A microscopic focus (2 × 2 mm) of a similar tumour nodule was seen in the left lobe. In one of the sections, the tumour showed mitotic count of 8/10 high power field (HPF) (~2 mm^2^), indicating a focus of DHGTC, a newly introduced entity by WHO, DHGTC (Figure 2b). The patient was given post-operative 100 mCi of oral radioactive iodine therapy (Iodine-131) and is under regular follow-up.

## Discussion

S/T PTC forms 1% to 3% of PTC [7]. WHO defines this subtype as having >50% solid, trabecular, or nested growth pattern [6]. Historically, associated with the Chernobyl nuclear accident, S/T PTC was thought to occur in children and young adults exposed to radiation. Later, it was found that this rare subtype can occur sporadically in adult patients without any radiation history, as seen in the current case [8]. Unlike other aggressive subtypes of PTC, the cytologic features of S/T PTC are challenging to discern preoperatively unless there is strong clinical suspicion.

Common cytologic patterns described in the literature include trabeculae, cohesive solid nests, syncytial fragments and three-dimensional clusters [7, 9]. Other possible patterns include microfollicles and scattered single cells (dyshesive) [10]. The current case showed predominantly trabecular arrangement. Intact trabecular fragments may be construed as papillae with intervening fibrovascular septa mimicking papillary cores (Figure 1a). Few of the clusters deciphered as papillaroid fragments with anatomical borders were solid, cohesive nests, as these were seen along with the trabecular clusters (Figure 1d). Microfollicles are usually fewer and represent abortive follicles seen on histology (Figure 1e and f). The predominance of microfollicular pattern causes difficulty in distinguishing this subtype from follicular subtype of PTC. A predominant microfollicular pattern was observed by Giorgadze *et al* [10] in 4/13 cases and Guleria *et al* [7] in 1/9 cases. Guleria *et al* [7] have mentioned endothelial cell lining in the trabecular nests in S/T PTC; however, few other studies mention this feature exclusive to poorly differentiated thyroid carcinoma (PDTC) [11]. We have noted endothelial cell lining in a few trabecular nests (Figure 1a, b and d) and believe this feature cannot be exclusive to PDTC.

Nuclear crowding, overlapping, grooving, INCI and chromatin clearing are seen to a variable extent in S/T PTC [2, 3, 7]. However, studies have found that the nuclei, in some cases, might appear round and darker [9, 10]. This forms another pitfall, with a possibility of rendering an alternate diagnosis including follicular neoplasm/suspicious of follicular neoplasm [10]. The current case also showed many areas with round, darker nuclei associated with microfollicles (Figure 1h). An important and close differential of S/T PTC is PDTC, especially in cytology [7]. PDTC may also show a similar trabecular/solid, cohesive pattern on cytology. However, classic nuclear features of PTC rule out PDTC [6]. The current case showed both nuclear grooves and an occasional INCI. Histologically, PDTC is characterised by increased mitotic figures (≥3/2 mm^2^) and/or necrosis [6]. These features are infrequently appreciated in cytology, making classic PTC nuclear features the essential reliable element to distinguish between S/T PTC and PDTC in cytology [7, 12].

WHO has introduced a new category called ‘DHGTC’, which shows features of papillary or follicular carcinoma with mitotic count ≥5/2 mm^2^ and/or necrosis [6]. DHGTC has an aggressive clinical outcome intermediate between that of well-differentiated thyroid carcinoma and anaplastic thyroid carcinoma [13]. The current case showed mitotic count 8/10 HPF (2 mm^2^), which was seen only in one section of the completely submitted tumour nodule. Vural *et al* [8] noted mitotic rate ≥5/10 HPF on histological sections of S/T PTC in 3/28 cases (10.7%), while Nikiforov *et al* [2] found mitotic rate 1–6/10 HPF in 5/20 cases (25%). Studies on cytological features of S/T PTC did not find mitotic figures [7, 10, 11]. It is worthwhile to note that in histological sections, increased mitosis with solid/insular architecture may lead to an erroneous diagnosis of PDTC, partially fulfilling the Turin consensus criteria. However, the presence of conventional nuclear features of PTC in such a case favours a diagnosis of DHGTC [6]. The presence of focal mitotic hotspots, as noted in the current case, highlights the importance of taking adequate sections of the tumour.

DHGTC may arise from high-grade progression of differentiated thyroid carcinomas (DTC), as evident in the current case or *de novo*. Progression from an existing DTC explains the prevalence of driver mutations in DHGTC which are also found in the corresponding DTC. In addition, late event mutations are responsible for the progression to high grade. Most of the DHGTC cases show *BRAF* p. V600E mutations with late changes of *TP53* and *TERT* promoter mutations [6]. Patients of DHGTC require intense treatment and active follow-up [13]. The 5-year disease-specific survival for DHGTC is 70% [14].

## Conclusion

PTC with its wide variety of subtypes may pose challenges in cytologic diagnosis. Alerting the surgeons about a possible aggressive subtype may help them in better planning of management. The present case highlights the salient features of S/T PTC in cytology and its close differential PDTC. The presence of classic nuclear features of PTC despite the presence of solid/insular cytoarchitecture and absence of necrosis should help in avoiding a diagnosis of PDTC in cytology. In surgical specimens, taking adequate sections is important to avoid missing a focus of DHGTC. Moreover, careful assessment of nuclear features of PTC in histological sections is of paramount importance in the case of S/T PTC with increased mitosis. The absence of the PTC nuclear features in such a case satisfies the diagnostic criteria of PDTC, while an unequivocal presence of PTC nuclear features points to a diagnosis of S/T PTC with a focus of DHGTC, as seen in the current case.

## Statement of informed consent

Informed consent was obtained from the patient about the possible publication of the case without revealing the personal details.

## Conflicts of interest

The authors have no conflicts of interest to declare.

## Funding

There was no funding received for publishing this case report.
